# “No PBL is better than online PBL”: Qualitative exploration regarding the perceived impact of online problem-based learning on nursing and medical students’ learning during COVID-19 lockdown

**DOI:** 10.21203/rs.3.rs-3296163/v1

**Published:** 2023-09-20

**Authors:** Pamella R Adongo, Joshua Epuitai, Joseph Luwaga Mpagi, Rebecca Nekaka, Ivan Lyagoba, Joseph Odula, Paul Oboth

**Affiliations:** Busitema University; Busitema University; Busitema University; Busitema University; Busitema University; Busitema University; Busitema University

**Keywords:** Online learning, problem-based learning, Uganda, COVID-19, learning and teaching

## Abstract

The COVID-19 pandemic and its restrictions increased the adoption of online learning even in low-income countries. The adoption of online teaching methods may have affected teaching and learning, particularly in settings where it was used for the first time. This study was conducted to explore the perceptions of medical and nursing students regarding the impact of online delivery of problem-based learning (PBL) on students learning and academic performance during COVID-19 imposed restrictions.

**Methods and materials:**

This was a qualitative study among fourth and fifth-year nursing and medical undergraduate students at Busitema University Faculty of Health Sciences. Four focused group discussions were conducted and the interviews focused on students’ perceptions, experiences, and attitudes toward the PBL process conducted online and its likely impact on their learning. Braun and Clarke’s thematic analysis was used for qualitative data analysis.

**Results:**

Four themes were identified that represented perceptions of online PBL on learning: transition to online learning; perceived benefits of online learning; limited learning and poor performance; and lost soft and practical skills. During the initial stages of introduction to online PBL learning, students transitioning to online had to adapt and familiarize themselves with online learning following the introduction of online learning. Students perceived that learning was less online compared to face-to-face sessions because of reduced learner engagement, concentration, motivation, peer-to-peer learning, and limited opportunities for practical sessions. Online learning was thought to increase students’ workload in the form of a number of assessments which was thought to reduce learning. Online tutorials were perceived to reduce the acquisition of soft skills like confidence, communication, leadership, and practical or clinical skills. While learning was thought to be less during online teaching, it was noted to allow continued learning during the lockdown, to be flexible, enhance self-drive and opportunity for work, solve infrastructure problems, and protect them from COVID-19 infection

**Conclusion:**

Generally, online learning enabled continuity and flexibility of learning. However, online PBL learning was perceived to be less engaging compared to traditional classroom-based PBL. Online PBL was seen to deter students from acquiring critical generic and clinical skills inherently found in traditional PBL. Innovative pedagogical measures should be adopted to avoid reduced learning noted in the online teaching methods to ensure the successful adoption of online teaching and learning in the post-COVID-19 era.

## Background

Problem based Learning (PBL) is a self-directed approach to learning which focuses on problem solving [1]. PBL was introduced to solve the lack of clinical competence among medical and nursing students[1]. Several medical schools have adopted PBL for training of health professionals [1].

COVID-19 pandemic and its related restrictions such as school closure, social distancing, and lockdown affected the teaching and learning process of students. COVID-19 led to drastic changes from traditional in-person method of teaching to online teaching. The transition to virtual learning modalities has been rife with challenges such as family distractions, timing of tutorials, lack of devices, and poor internet which might have the performance of students [2]. Studies have noted that conflicting evidence regarding the impact of switching to online learning on the performance of students [3, 4]. In one study, the academic performance was significantly lower in online PBL compared to face-to-face tutorials including in all areas of participation, communication, preparation, group skills, and critical thinking [3]. In contrary, mean PBL scores were significantly higher among students who were doing online PBL compared to face-to-face PBL sessions[4].

Studies have attempted to explore the experiences of students and faculty with the adoption of virtual learning strategies [4]. Currently, there is limited scholarship regarding the impact of online delivery of PBL on the academic performance of students. Our study will provide key findings regarding student’s performance and perceptions of the effect of online tutorials during COVID-19 pandemic on their performance. The purpose of conducting the study was to explore perceptions of students regarding the benefits of online PBL and its associated impact on the learning experience of students in Busitema University.

## Methods and Materials

### Study design and setting

This was a qualitative phenomenological study which was conducted to explore experiences and perceptions of students regarding learning online during COVID-19 lockdown. The study was conducted in Busitema University Faculty of Health Sciences (BUFHS). Busitema University Faculty of Health Sciences offers undergraduate and graduate programs including Bachelor Medicine and Surgery, Bachelor of Nursing Science and Bachelor of Anesthesia. The faculty has about 450 students. In Busitema University Faculty of Health Sciences, PBL is primarily mode of teaching in basic and clinical years. Following suspension of classes from February 2021 to May 2021 due to the outbreak of COVID-19 disease, teaching shifted from the conventional physical to virtual learning. The teaching methods during lockdown including online lectures, tutorials, and seminars, while some assessments were done online.

### Study Population

The study population comprised of nursing and medicine students studying Bachelors of Nursing Science and Bachelors of Medicine and Surgery programs respectively. Students in the fourth and fifth year of study were included as they had experiences of online and physical delivery of PBL. Students who did not consent were not included in the study. Purposive sampling was used to select participants based on student leadership roles, year of study and interpersonal skills. Sample size in the qualitative study was based on the principle of data saturation when no new information was reached [5].

### Data collection tool and procedure

Interviewer-guide was used to interview to collect data. Interviews were conducted by IL & JO, who were final year nursing students. The study used focused-group discussions (FGDs) to interview students since FGDs would allow students to relate and recall, during the discussion, their experiences with online teaching method. Open-ended questions in the interviewer guide included exploration of the experiences, perceptions, attitudes and thoughts towards online delivery of PBL during the COVID-19 lockdown. Students were asked about the effect of online learning on their learning and performance and suggestions of how their experience of learning online could be improved. Data collection was conducted in October 2022.

### Data analysis

Audio recording was transcribed verbatim. Thematic analysis, as described by Brauna and Clarke, was used for analyzing qualitative data [5]. Thematic analysis was done through reading and re-reading of the transcript for several times, identification of codes, categories, and themes, which describe the data.

### Ethical Consideration

The study obtained ethical approval from BUFHS Research and Ethics Committee (BUFHS-2022-17). Written informed consent was obtained from the study participants.

## Results

### Description of the participants

This study involved conducting four FGDs each comprising seven participants, fourteen (n = 14) nursing students and twenty (n = 20) medicine students. The age difference was between 22 to 30 years. Fifth year students were n = 21 whereas fourth year students were n = 14.

### Perceptions regarding impact of online PBL during COVID-19 lockdown on academic learning

**Figure 1:**
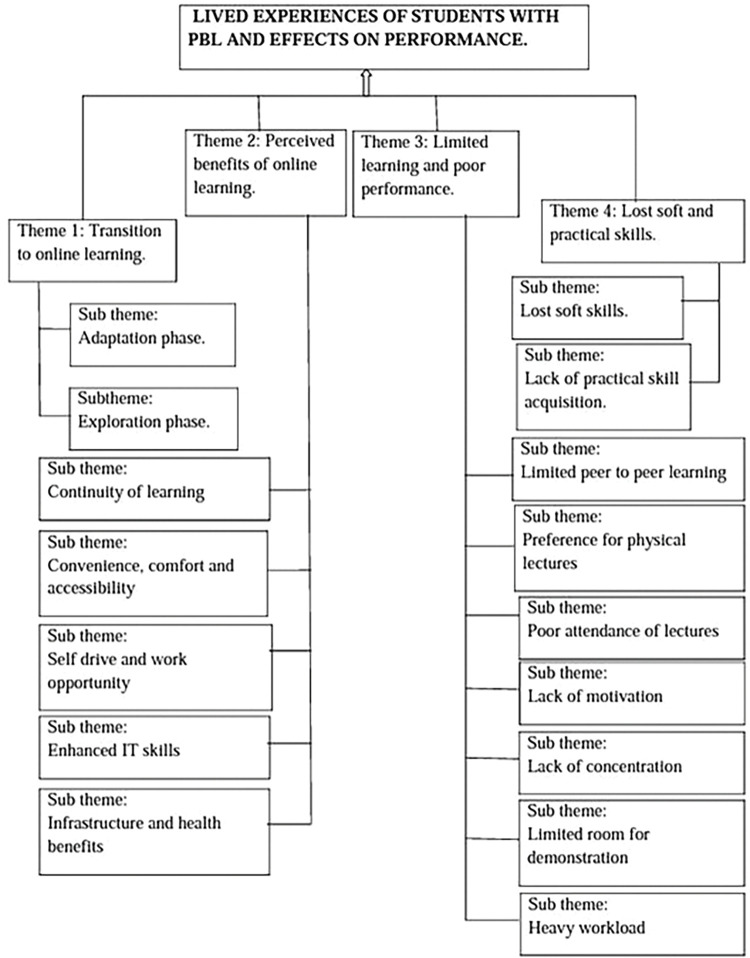
Perceptions of students regarding impact of COVID-19 lockdown on academic learning

The study explored the impact of online PBL during COVID-19 on academic learning of undergraduate nursing and medical students. Overall, four themes described the impact of online PBL on academic learning: transition to online learning, perceived benefits of online learning, limited learning and poor performance and lost soft and practical skills (Fig. 1).

#### Theme 1: Transition to online learning

##### 1.1 Adaptation phase

COVID-19 enforced restriction of the lockdown which forced most of the teaching institution to switch to online PBL strategy of pedagogy. Students and lectures had no prior experience of online learning as the majority of them interfaced with the online learning for the first time. Students reported poor experience in the early phases of online learning which forced them to adapt in the process of transitioning to online learning.

*“My experience at first it was not really good, but as time went on, I kept on adjusting ............. online really happened for the first time of its kind because of the lockdown.”* (FGD-3, Fourth year nursing student).

*“So, for me, I had experiences with tutorials online but I think as they said, I think we are transitioning…….”*(FGD-2, year four nursing student).

##### 1.2. Familiarization phase.

Transition to online learning was marked with exploration of online platforms. Students had to learn for the first time about how to use Zoom and Learning management system. The exploration phase was reflected with less knowledge about how to use the online platforms as students and lecturers were getting familiar with the online learning platforms.

*“….then online learning came with adjusting. It came with needing to shift from physical lectures we got used to from primary school to the online way. So, it needed me to learn more about Zoom, this Google Meet…”* (FGD-4, year five medical student).

“Sometimes, since it was a new mode of learning, some of us did not know Zoom. So, you find yourself, you don’t know where to click, like not to be heard [to mute microphone]. So that also became a challenge like for me personally when it was beginning” (FGD-1, year five medical student).

#### Theme 2: Perceived benefits of online learning

##### 2.1 Continuity of learning

Although most of the students recounted that they did not learn much from online compared to physical lectures, some admitted that online teaching enabled them to at least continue learning even when schools were closed during the lockdown. However, some noted that even this perceived advantage was watered down because teaching was repeated when the university was reopened which stopped them from progressing to the next academic semester and or year.

*“…..So, I think the only advantage I got from doing online was that we were not totally left behind in terms of our course. At least, we did not stay home completely without studying”* (FGD-4, year five medical student).

*“Personally, I am grateful to the online system because I am trying to relate if there was nothing for us and we just wait for the lockdown to be lifted and come back. I don’t know where we would be right now….so, the online system came in as a savior….”*(FGD-2, year four nursing student).

“….I think online learning especially during COVID did not do much to me……because if online learning had maybe added something, we would not be having fifth years here now. They would have finished……when we were at home for online, when we came back, we had to redo everything afresh…. (FGD-1, year five medical student).

##### 2.2 Flexibility.

Students noted that online learning was convenient and comfortable for them. Online learning enabled them to attend learning sessions remotely in the comfort of their homes. Students also thought that online learning was convenient for lecturers as it enabled them to teach even when they were away from the university premises. Online learning eliminated challenges of infrastructure including limited space for lectures as well as inconveniences of weather and tendencies for late coming for lectures.

*“For me, the advantage was learning in the comfort of where I stay, that was the best advantage.”* (FGD-4, year five medical student).

*“…. So online is really good. It is convenient. Most times the lecturers are not around, and so they are still able to cover them instead of missing them in the name of face to face”*. (FGD-2, year four nursing student)

Additionally, students were able to conveniently access all the learning materials. Online learning, enabled students who did not understand concepts in the lecture, to revisit the recorded session at their own time of convenience. Besides accessibility of learning materials from the University, students were able to conveniently attend several learning sessions from different places outside the confines of the university.

*“PBL at the time when it was transferred online, we were able to get all information. Our lecturers would put all the information and we were able to access it at anytime and anywhere we are free.”* (FGD-1, year five medical student).

*“Online learning has come with more of interacting with many people, international, even in our zoom lectures people from different universities would attend. Even me, I would attend sessions of other universities….”* (FGD-4, year five medicine student).

##### 2.3 Self-drive & work opportunities

Students, who had formal employment, valued the flexibility that online learning offered as it enabled them to continue working while studying. Students also added that online learning indirectly taught them to be self-motivated to attend and concentrate during the lecture as it no longer needed the peering eye of the lecturer to be the motivation to concentrate during the lecture as it is in physical lectures.

*“…one, it gave me a chance I think I used to work at night and lectures are always during day, and I would work like 6 hours of the night then lectures during day. The lecture is at 8 am, you have just put your head down [and you have to attend the lecture]. That is even one of the contributing factors to depression…”* (FGD-4, year five medical student).

*“For me, I think online learning is based on the concept that a university student is mature and also must be honest with himself. For me, online learning gave me a chance to assess my own self in honesty. If they have said there is a lecture, will I sit and actually be attentive to it…”* (FGD-4, year five medical student).

##### 2.4 Enhanced information technology skills.

Initially, students did not know much about information technology in general. As a result, students faced challenges when using online learning platforms including difficulty maintaining proper online learning etiquette, logging into the online platform, or active participation in the online platforms. However, students observed that online learning enabled them to develop new skills in information communication technology (ICT).

*“Some of us, we are trying to improve the ICT. I did not know Zoom exists, LMS, and now I have learnt new things and they are so good.”* (FGD-2, year four nursing student).

Well as much as we had a lot of challenges, but there were new learning. I for one, I learned how to use digital system of learning. I really had no idea. I had never used Zoom before….my learning was not enhanced, I mean in [academic] performance but I also learnt new things, more so of internet…” (FGD-4, year five medical student).

##### 2.5 Infrastructure and health benefits

Students noted that online learning prevented the spread of COVID-19 as it reduced social interaction. Students also highlighted that online teaching reduced the need for physical infrastructure especially in a resource constrained setting with limited space for lectures.

*“The other positive with it, it allowed curbing down the spread of COVID 19 because you could or people would not have to come to a single place to deliver or attend the lecture, so it would help in preventing the spread of COVID 19”* (FGD-3, year four nursing student).

*“So online teaching in our university is good because we don’t have infrastructure, we don’t have where to sit….. so that’s where online teaching comes in, because you can sit anywhere you want and attend the lectures”* (FGD-2, year four nursing student).

#### Theme 3: Limited learning and poor performance.

Although some students remarked that their performance improved or remained the same following the introduction of online learning, overall, the majority of the students thought that most of the learning was less or even worse during online compared to face to face. Online lectures, tutorials, seminars and assessment were perceived to be of little benefit to students. Learning and academic performance during online were perceived to have deteriorated for a number of reasons including limited peer to peer learning, preference for physical lectures/tutorials, lack of engagement, motivation, concentration, demonstration/clinical placement and heavy workload.

##### 3.1 Limited peer to peer learning.

Students were not able to meet and discuss because of the lockdown restrictions of social interactions. Holding discussions online among students were not feasible because of the costs of internet, procuring Zoom license, network challenges and the limited quality of discussions that were online. The limited opportunity to hold a physical discussion among students was perceived to greatly affect their learning experience and ultimately their academic performance.

*“Some people really benefited in discussions. So, when online started, were divided and we couldn’t hold online discussion…..so, online teaching affected people who depended on discussion to improve their learning and performance.” (*FGD-2, *year four nursing student)*.

#### 3.2 Preference for physical lectures

Physical lectures were thought to be more beneficial to students as it had room to ask questions. Physical lectures is perceived to be more easily understood partly because it had the visual element which enabled to see the non-verbal demonstrations made by the lecturer.

*“Personally, I faced the same challenge [low performance] in that the physical lectures, you get more time to ask questions where you have not understood. But as we have said with online lectures, you get network issues, the data is done, you are off….”* (FGD-1, year five medicine student).

*“….and another thing, a lecture, which is delivered physically is not the same way like the online lecture. Like you benefit more from a physical lecture more than an online lecture.”* (FGD-1, year five medicine student).

*“…online affected me negatively…I only passed with survival [borderline] marks…I always understand when someone is explaining to me and it gets to memory when I see that person explaining to me something but for the case of online, I would even try to remember something in the exam, it just comes partially but then it doesn’t come. At the end of it all I just had fragmented information.”* (FGD-1, year five medical student).

##### 3.3 Reduced learner engagement

Students thought that learning during online was negatively affected by poor attendance of lectures. Furthermore, attendance of online lectures became a routine where the majority of the students would log in not to actively attend the lecture but for the sake of registering their attendance.

*“My learning was greatly affected negatively. I just mentioned that I chopped lectures like never before. Like I did not have the drive to learn. I remember that I had started involving myself in some business so I skipped almost 60% of the lectures and that greatly affected my results when we came back for examinations.”* (FGD, year four nursing student).

*“To me, during the time of online learning, I used to attend just because I had to and for purposes of registration…..so to do with the performance, it generally, somehow affected [the performance]”* (FGD-1, year five medical student).

*“….all I could do was log in, put the phone there and continue doing my own things….as a student you have not benefited much from it. So, that for my case affected a lot of learning process at home.”* (FGD-2, year four nursing student).

##### 3.4. Lack of motivation.

Students seemed to lack intrinsic and extrinsic motivation to learn, this ultimately affected their learning and academic performance. Because no one would be supervising them, students were no longer motivated to read and prepare for their tutorials, as they would just read from gadgets. This was in contrast to physical tutorials where students would always widely read in preparation to present their work during the tutorial. Physical isolation meant that students were not nudged by their peers to read either, while the home environment deterred them from reading.

*“Tutorials during online were not really effective….I mean I wouldn’t challenge myself very well to go and read and present on Zoom….If you’re physically present you take more time to understand the concept, but on Zoom, me I stopped reading for tutorials….I now go to Google to look for answers. I just wait and read them.”* (FGD-4, year five medical student).

*“To me I think, the online delivery affected my performance to a greater extent…..at school, you get guts of reading from your fellows. Like you see your fellows reading and you say, let me read. But at home, people are watching TV and they are laughing, they are not in the reading mood.”* (FGD-1, year five medicine student)

##### 3.5 Lack of concentration

Students were not able to learn effectively related to lack of concentration during lecture and tutorials. Online learning removed the pressures of a lecture and tutorial and the incentive to concentrate during the lecture, while others failed to see the relevance of online lectures.

“Me personally, if you say an online lecture, I will not concentrate up to now. I don’t give it much attention like if it was a physical lecture. Sometimes, I can just login and do other things. Like my mind is elsewhere.” (FGD-1, year five medical student).

*“You concentrate more if you are face to face other than online where you know that the lecturer is 100Km the other way….”* (FGD-1, year five medical student).

“It wasn’t as serious as you could be at school. It could not give you that pressure or tension of going to class and finishing your assignments according to the deadline. I never got that feeling of it being serious to me like the physical lectures.” (FGD-3, year four nursing student).

##### 3.6. Limited room for demonstration/clinical placement

Learning during online was perceived to greatly suffer from limited room for practical sessions, demonstration and clinical placement. Students valued the importance of touching and visual amenities in facilitating the learning process.

*“.......you find that we didn’t do practical during that time and at that particular point, it would be much easier if you were doing practical with theory so that you can coordinate the practical and the theory........... It looks like I am doing a practical in isolation with theory and it was quite hard for me to link the two.”* (FGD-1, year five medical student).

*“….we are medical students and we learn by interacting with patients like we learn through demonstrations and interacting with each other and then our seniors but with online PBL, most of those things you can’t access them. So, learning is very affected.”* (FGD-3, year four nursing student).

##### 3.7. Heavy workload

During COVID-19 lockdown, several e-learning activities were created on learning management systems to keep the students engaged in the learning process. Students felt that the e-activities and assessments were too many which negatively affected their ability to independently read. The heavy workload forced students to engage in rote learning, plagiarism, copy and paste practice without taking effort to comprehend the information.

“The online assessments were many and we had to meet the deadlines. So, basically learning was very minimal because you could just do just for the sake of meeting deadlines.” (FGD-1, year five medical student).

*“This thing [online lectures] never felt like it was serious….it wasn’t as serious as you could be at school. It could not give you that pressure or tension of going to class and finishing your assignments according to the deadline.”* (FGD-3, year four nursing student).

“….we had online tests and online discussion forum. We would just go and copy the whole information in the textbook and put it there. Now, when it came to physical where we were supposed to answer questions, you find yourself unable to perform because you were not able to comprehend the information which you had put there…..” (FGD-1, year five medical student).

#### Theme 4: Lost soft and practical skills

##### 4.1 Lost soft skills

Many students believed that online PBL made them lose their soft skills such as social life, public speaking, leadership, smartness, time management and confidence. Students noted that while studying online such skills were no longer necessary because one would attend online sessions without the need to exhibit proper dress code as it was required in physical tutorials. Some students remarked that face to face PBL enabled them acquire competence and confidence in public speaking which skills were not possible in online tutorials.

*“…….my comment is that no PBL is better than online PBL for three additional things. Number one besides the academic part of life, there is a lot we learn from tutorials: things like social life is being improved, things like smartness, time keeping, confidence. So, PBL online doesn’t give room for a person like me who came ready express themselves….”* (FGD-4, year five medicine student).

*“.... problem based learning made me one of the competent and confident student........... standing in front of others and then you speak out what you have confidently without fear or favour ......... it gave me that energy to speak out what I had and now I think I can now apply it somewhere else”* (FGD-3, year four nursing student).

“I wouldn’t say that I enjoyed it because it was very different generally. Like what we usually have in our room, all of us in one room. The feeling you get when attending or presenting to a group.” (FGD-1, year five medical student)

##### 4.2 Lack of practical skill acquisition

Furthermore, students also noted that online PBL did not support practical skill acquisition.

*“So, there’s a practical aspect of it where maybe someone has to and clerk a patient and then come and present. Something like that. Someone told us to go and examine abdomens of guys in the village there. It doesn’t add up. So, PBL online doesn’t give room for practical aspect. But even without patients, there are some practical aspects that are really involved even the students themselves. Maybe something will be shown to us using a student. So, [online] PBL doesn’t give room for that.”* (FGD-4, year five medical student).

## Discussion

This study explored a deeper understanding of the perceptions of students about online learning and its effects on academic performance. In the early stages of the lockdown, students had to transition from physical learning they were familiar with to online learning, and therefore, had to adapt and become familiar with learning online. Students recounted benefits offered by online PBL during COVID-19 lockdown but also pointed out its limitations especially on reduced learning and academic performance during online teaching. The findings from this study offer important insights critical to the use of online platforms for teaching and learning after the lifting of COVID-19 restrictions.

Online learning in health professions education inheres transformational benefits especially in settings with limited infrastructure and human resource [6]. However, adoption of online learning for medical and nursing educations in sub-Saharan Africa has been slow and unsustainable [7]. In low income countries, COVID-19 pandemic marked a watershed moment characterized with rapid adoption of online teaching in the training of health professions [8]. As a result, most of the students in our study interfaced with online learning for the first time, and had no prior knowledge of online learning, a finding which is consistent with previous studies[9–11]. Consequently, as part of transitioning from physical to online methods of teaching, students had to adapt, learn and become familiar with using online learning. Similarly, students thought that the university was in the transition stage in regards to use of online learning, and highlighted teething problems with infrastructure, mode of delivery online, and expertise in using the new pedagogical approach.

Contrary to findings that online teaching methods enhanced learning during COVID-19, most of the students in our setting thought that learning was less, insignificant and not beneficial during online compared to face-to-face sessions. Like the previous studies [12], learning was negatively affected by lack of learner engagement and concentration amidst myriad of external and internal distractions from home environment, demands of work, and lack of supervision. Secondly, social isolation removed the safety net of academic discussion groups among students which ultimately reduced their ability to easily comprehend information. Students in online tutorials stopped reading in preparation to present their work in tutorials as the lack of face-to-face interactions favored malpractices of reading directly from their reference learning materials. Learning occurs in the context of social interactions and ability of students to imitate each other and adopt behaviors beneficial for learning. Lockdown restrictions antagonized social interactions among students and their peers which altogether was thought to reduce their motivation and peer to peer learning. Although assessment drives learning, the use of a number of assignments, tests and lectures during online learning was thought to inhibit learning through promoting rote memorization, examination malpractices and plagiarism. Lastly, learning in online PBL suffered from limited opportunities for students to engage in practical and demonstration sessions. Ultimately, students thought that limited learning translated to poor academic performance. Teachers of medical education need to embrace innovative teaching methods including use of short lectures and e-learning activities that maximize the transformative role of online teaching in medical and nursing education.

Although students thought that learning online was less compared to physical, students admitted a number of benefits associated with online PBL. As online PBL was introduced when institutions of learning were closed because of COVID-19, students believed that online learning enabled them to continue with their studies. This findings are consistent with other studies that cited convenience and accessibility as the benefits of online learning [12]. In our setting, online learning was thought to be convenient for lectures as well as it enabled them to teach remotely from the university premises, a finding which concurs with the projected benefit of online teaching in areas of limited human resource personnel [8]. Relatedly, online learning was perceived to solve issues of space and infrastructure such as lecture halls. The flexibility of online learning enabled students to balance work and studies. COVID-19 induced online learning was thought to increase the ICT skills of students particularly given the low use of internet and ICT equipment in our setting.

Previous studies have underscored the role of PBL in promoting acquisition of generic skills [1, 13]. PBL confers desirable generic skills including skills in leadership, communication, public speaking, social congruence, active listening, interpersonal relations, team work and self-directed learning [1, 13]. In our study, students thought that online tutorial does not provide them with a platform to gain self-confidence, communication and leadership skills. This concurs with a study which reported that students in online tutorial performed poorly in all five areas of assessment compared to those in face-to-face tutorials [14]. Students in online tutorials performed poorly in domains of participation, communication, preparation, critical thinking and group skills [14]. Poor acquisition of public speaking skills maybe attributable to the fact that online learning removes need for social etiquette and the associated pressure of presenting in front of others. Besides problems of internet connectivity and distractions, poor acquisition of interpersonal skills among other generic skills could be attributed to lack of a strong group cohesion and connectedness in online compared to physical lectures [14]. Ultimately, reduced soft skills in online PBL suggests that its introduction may threaten the gains made from integration of PBL in institutions of learning [1].

## Conclusion

In the initial stage, following introduction of online learning, students had to transition to the new way of teaching which manifested inform of adaptation and familiarization with the online learning platforms. Online learning was perceived to be less during online PBL compared to face-to-face sessions. Reduced learning in online was attributed to lack of student engagement, concentration, motivation, peer-peer learning, limited practical sessions and heavy workload. Students thought that online tutorials did not provide them with a platform to enhance their generic skills of public speaking, leadership, communication skills and self-confidence. Students also noted that online learning affected their acquisition of clinical and practical skills. Although learning was less, students admitted that online teaching allowed continuation of learning, promoted self-drive and opportunity to continue working, enhanced their ICT skills, protected them from COVID-19 infection and reduced problems of infrastructure for teaching space.

## Data Availability

The additional data and materials can be accessed from the corresponding author on a reasonable request
